# Insights into estrogen impact in oral health & microbiome in COVID-19

**DOI:** 10.1186/s12866-023-03149-5

**Published:** 2024-01-20

**Authors:** Rubén Alberto Bayardo-González, Marcela Peña-Rodríguez, Ana Laura Pereira-Suárez, Alina Xcaret Rubio-Sánchez, Mariel García-Chagollán, Daniel Natividad Valenzuela-Orozco, Melida del Rosario Lizarazo-Taborda, Jesús Mora-Mora, Natali Vega-Magaña

**Affiliations:** 1 Programa de Doctorado en Microbiología Médica, Centro Universitario de Ciencias de La Salud (CUCS) Universidad de Guadalajara, Guadalajara, Jalisco México; 2 Laboratorio de Diagnóstico de Enfermedades Emergentes y Reemergentes (LaDEER), CUCS, Universidad de Guadalajara, Guadalajara, Jalisco México; 3 Instituto de Investigación en Ciencias Biomédicas (IICB), CUCS, Universidad de Guadalajara, Sierra Mojada 950, Colonia Independencia Oriente, Guadalajara, Jalisco CP. 44340 México; 4 Programa de Maestría en Microbiología Médica, CUCS, Universidad de Guadalajara, Guadalajara, Jalisco México; 5 Programa de Licenciatura en Cirujano Dentista, CUCS, Universidad de Guadalajara, Guadalajara, Jalisco México

**Keywords:** COVID-19, Microbiome, Cytokines, Inflammation, Women-s health

## Abstract

**Background:**

COVID-19 emerged in late 2019 and has occasioned more than 765 millions cumulative cases and 6.9 millions of deaths globally. Notably, around 70% of patients with severe COVID-19 are men. Therefore, it is to be presumed that women have a hormonal protector factor in inflammation and ACE2 expression. On the other hand, oral health status, and local microbiome can be key factors to respiratory viral infections control. Nevertheless, it has been poorly investigated. In our study 20 premenopausal, 18 postmenopausal and 22 men with COVID-19 were included. Oral health status, viral load, lingual ACE2 expression, as well as microbiome, estrogens and cytokines in saliva were analyzed.

**Results:**

Our results showed a lower expression of ACE2 in tongue cells of postmenopausal compared with premenopausal (*p* = 0.05), and a strong negative correlation between saliva estrogen and viral load (*r* = -0.76; *p* = 0.001). Respect to IFN-γ (*p* = 0.05), IL-1β, TNF-α, IL-18, and IL-23 levels were increased in postmenopausal. Oral microbiome signature of premenopausal was characterized by *Prevotella melaninogenica* (Log2 = 26.68; *p* = 1.34e-10)*, Haemophilus* (Log2 = 23.99; *p* = 2.96e-9)*,* and *Alloprevotella* (Log2 = 7.92; *p* = 0.0001)*.* On the other hand, *Leptotrichia* (Log2 = -18.74; *p* = 0.001)*, Tanerella* (Log2 = -17.08; *p* = 0.004)*,* and *Clostridiales* (Log2 = -2.88; *p* = 0.04) represented the poor oral health group compared with the adequate group which was enriched with the commensal microorganism *Neisseria perflava* (Log2 = 26.70; *p* = 1.74e-7)*.* Furthermore, the high viral load group was characterized by *Prevotella nanceiensis* (Log2 = 19.60; *p* = 6.06e-8)*, Prevotella melaninogenica* (Log2 = 21.45; *p* = 9.59e-6)*, Alloprevotella* (Log2 = 23.50; *p* = 2.70e-7) and bacteria from the red complex *Porphyromonas endodentalis* (Log2 = 21.97; *p* = 1.38e-7)*.*

**Conclusions:**

Postmenopausal and men have a poor oral health status which could be related to a detrimental progression of COVID-19 also linked to a lower expression of ACE2, lower saliva estrogen levels and oral dysbiosis. Nevertheless, functional studies are required for a deeper knowledge.

**Supplementary Information:**

The online version contains supplementary material available at 10.1186/s12866-023-03149-5.

## Background

The pandemic of COVID-19 spread rapidly worldwide and caused more than 6 million deaths [1]. Reports described that older adults, postmenopausal, and men are at increased risk of developing severe symptomatology and mortality compared to young women. As well obesity, high pressure, and bad general health status play an essential role in increasing mortality [2]. Sex disparity could be a key factor in the progression of COVID-19. Whereas incidence of SARS-CoV-2 infection are similar in men and women; on the contrary, men have a severe outcome disease, and a higher mortality rate compared to women [3, 4]. These differences could be linkage to female hormone levels, sex chromosomes in particular X-linked genes escaping silencing that can influence expression of *ACE2*, *TLR7,* and *TLR8* genes [5–7].

Oral cavity is an entry pathway for SARS-CoV-2. This virus can enter into the cell via the Angiotensin-Converting Enzyme type 2 (ACE2) receptor, which is expressed in the oro-nasal mucosa, lungs, heart, kidney, and vessels. ACE2 is an important molecule to deactivate the detrimental effects of the Renin- Angiotensin System (RAS) as vasoconstriction, sodium retention and the increase of blood pressure. SARS-CoV-2 infection can downregulate the protective functions of ACE2 [8]. Moreover, this virus can lead to a cytokine storm orchestrated by the innate immune system and the inability to clear the infection by the adaptive immune system [9].

On the other hand, estrogens have a beneficial effect on the upper and lower airways, hence the increase of nasal mucus which contains lactoferrin, IgA, IgG and mucins that have an antimicrobial effect [2]. Estrogens also promote an enhancement of the innate and adaptive immune system increasing macrophages, dendritic cells and natural killer activity; as well as the diminishment of inflammatory cytokines like IL-1, TNF-α, and IL-6; and promote interferon type 1 response [10, 11]. Subsequently, sex hormones, especially estrogens interfere with the sexual dimorphism reported in the immune response and microbiota composition [12–14].

Broadly speaking, local chronic inflammatory states caused by a lack of oral health or periodontal disease could lead to deficient immune response. Furthermore, SARS-CoV2 infection can cause epithelial injuries and alter the oral microbiome in patients, increasing inflammation-inducing pathobionts [15, 16], which are related to symptom duration and can lead to secondary bacterial infections [17]. The oral epithelial cells are capable of synthesizing a wide variety of cytokines; hence injuries can result in an environment with increased oral proinflammatory cytokines [15] that perpetuate microbiota dysbiosis and mucosal injury.

The aim of this study was to evaluate the role of estrogen in the modulation of oral inflammatory cytokines, oral microbiome and their impact in disease severity and oral health in premenopausal women with SARS-CoV-2 infection.

## Results

### Demographic, clinical characteristics and oral health status

Sixty subjects were included in the study and were divided into three groups, premenopausal (*n* = 20), postmenopausal (*n* = 18) and men (*n* = 22). Demographic and clinical data are described in Table 1. Significant differences were found in age (*p* = 0.0001), weight (*p* = 0.0004), height (*p* = 0.0001), and serum estradiol levels between groups (*p* = 0.0001). In contrast, salivary estradiol levels only showed a tendency to decrease in postmenopausal and men groups (*p* = 0.1630). On the other hand, when we analyzed comorbidities, we observed that postmenopausal women had a higher prevalence of hypertension, diabetes and obesity (*p* = 0.0238). Body mass index and SARS-CoV-2 viral load did not show statistical differences (Table 1). Oral health status was evaluated through DMF-T and Oral Hygiene Index. The postmenopausal group had the worst oral health status (*p* = 0.01) according to the DMF-T index (7.61 ± 3.27) compared with the premenopausal (4.61 ± 4.03) and men groups (4.5 ± 3). However, when the presence of caries was evaluated alone among the groups, men had the poorest oral status compared with the premenopausal and postmenopausal groups (*p* = 0.0483). According to the oral hygiene index (OHI-S), the three groups presented moderate oral hygiene. Nonetheless, when the Modified Gingival Index was evaluated, the postmenopausal women presented higher gingival inflammation compared to the premenopausal and men groups. No difference was observed among the number of patients that referred to bleeding when toothbrushing (Table 1). Symptoms are enlisted in Table S1.

**Table 1:** Demographic, clinical and oral health status data

Variables	Premenopausal *n* = 20	Postmenopausal *n* = 18	Men *n* = 22	*P value*
Age	33.55 ± 7.14	56.56 ± 10.25	37.55 ± 10.96	0.0001*
Weight (kg)	73.45 ± 15.95	67.94 ± 13.83	88.95 ± 18.38	0.0004*
Height (m)	1.62 ± 0.06	1.55 ± 0.05	1.75 ± 0.06	0.0001*
Serum estrogen (pg/mL)	88 ± 84	46 ± 56	25 ± 8	0.0001*
Saliva estrogen (pg/mL)	1.19 ± 0.49	0.98 ± 0.42	0.92 ± 0.33	0.1630
**Comorbidities**				
Yes (%)	7 (35)	14 (77.8)	10 (45.5)	0.0238 +
No (%)	13 (65)	4 (22.2)	12 (54.5)	
Hypertension	-	4 (28.6)	2 (20)	
Diabetes	1 (14.3)	3 (21.4)	1 (10)	
Obesity	6 (85.7)	7 (50)	7 (70)	
**Body Mass Index (%)**				
Normal Weight	7 (35)	4 (22.2)	3(13.6)	0.4459
Overweight	7 (35)	7 (38.8)	12 (54.5)	
Obesity	6 (30)	7 (38.8)	7 (31.9)	
**SARS-CoV-2**				
Viral Load (copies/mL)	8.24 × 10^6^ ± 16 × 10^6^	10.4 × 10^6^ ± 14 × 10^6^	4.9 × 10^6^ ± 10 × 10^7^	0.7225
High (> 100,000 copies/mL)	15(75%)	11(61.1%)	15(68.2%)	
Moderate (10,000 -100,000 copies/mL)	2(10%)	5(27.8%)	4(18.2%)	
No detected^a^	3(15%)	2(11.1)	3(13.6%)	
**Oral Health**				
**DMF-T**	4.6 ± 4.03	7.6 ± 3.27	4.45 ± 3	0.01⤒
**CAVITY CARIES (D component)**	1.4 ± 2	0.88 ± 1.77	2.09 ± 1.84	0.0483
**OHI-S**	1.12 ± 0.88	1.52 ± 1.21	1.55 ± 0.79	0.2983

Comparison by groups: + premenopausal women vs postmenopausal women

The Chi square test, ⤒ one-way ANOVA and *Kruskal–Wallis tests were employed for statistical analysis

^a^Patients “No detected” had a positive SARS-CoV-2 antigen test

### Lingual ACE2 expression

**Fig. 1:**
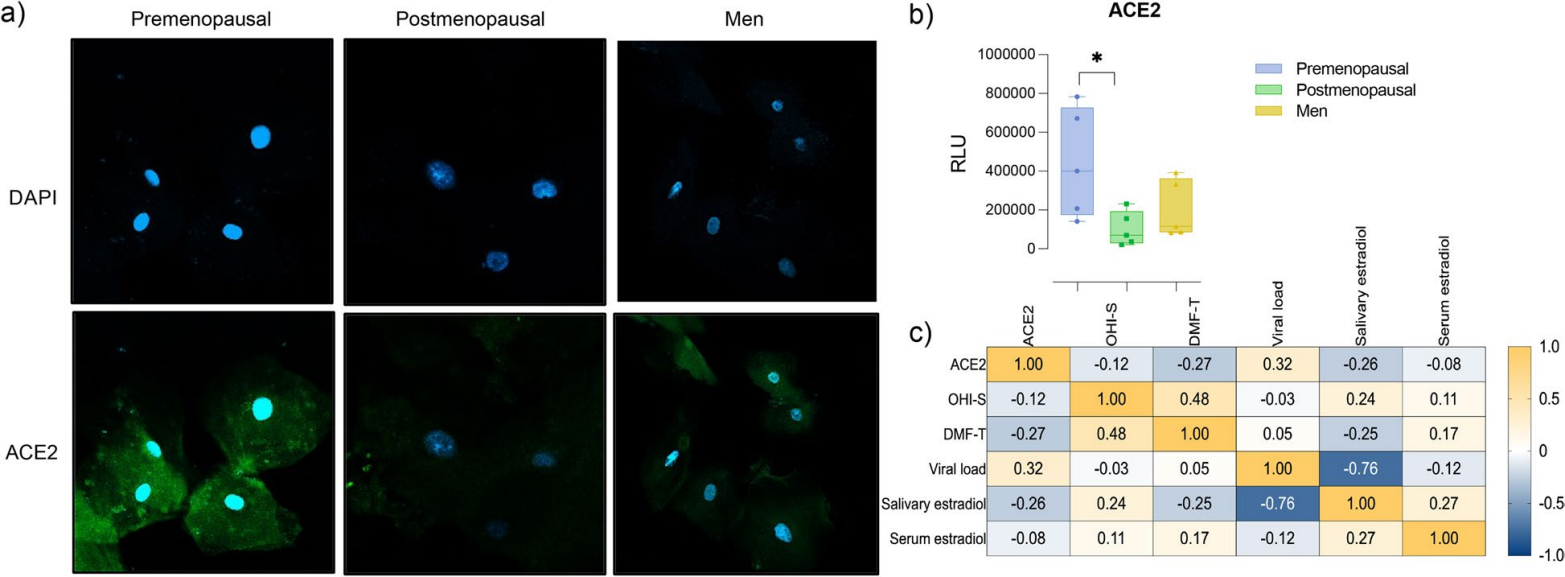
Lingual ACE2 expression and correlations. **a** representative images of ACE2 expression in tongue cells of premenopausal, postmenopausal and men by immunofluorescence. **b** Relative light units analysis of ACE2 expression. **c** Spearman correlation heatmap of ACE2, OHI-S, DMF-T, viral load, saliva estrogen, and serum estrogen levels. *p* values ≤ 0.05 were considered significant and *r* = 0.4 cut off. *ACE2: Angiotensin converting enzyme 2; OHI: oral hygiene index; DMF: Decay missing filled*

In order to analyze ACE2 expression in the oral cavity, we performed a lingual swab, and studied cells by Immunofluorescence. In Fig. 1a we show representative images of cells stained and visualized by a confocal microscope, as well as, the relative light units analysis (Fig. 1b). As we expected, ACE2 expression was at the cell surface, but also in an intracellular way. Interestingly, cells from premenopausal women had the highest expression of ACE2, in comparison to postmenopausal (*p* = 0.05), and men groups. Additionally, we performed correlation analysis of ACE2 with OHI-S, DMF-T, viral load, and estrogen levels in serum and saliva. Were found as expected a positive correlation between OHI-S and DMF-T (*r* = 0.48; *p* = 0.035); and an interesting negative correlation between estrogen saliva levels and ACE2 expression (*r* = 0.76; *p* = 0.001) (Fig. 1c).

### Proinflammatory cytokines in saliva

**Fig. 2:**
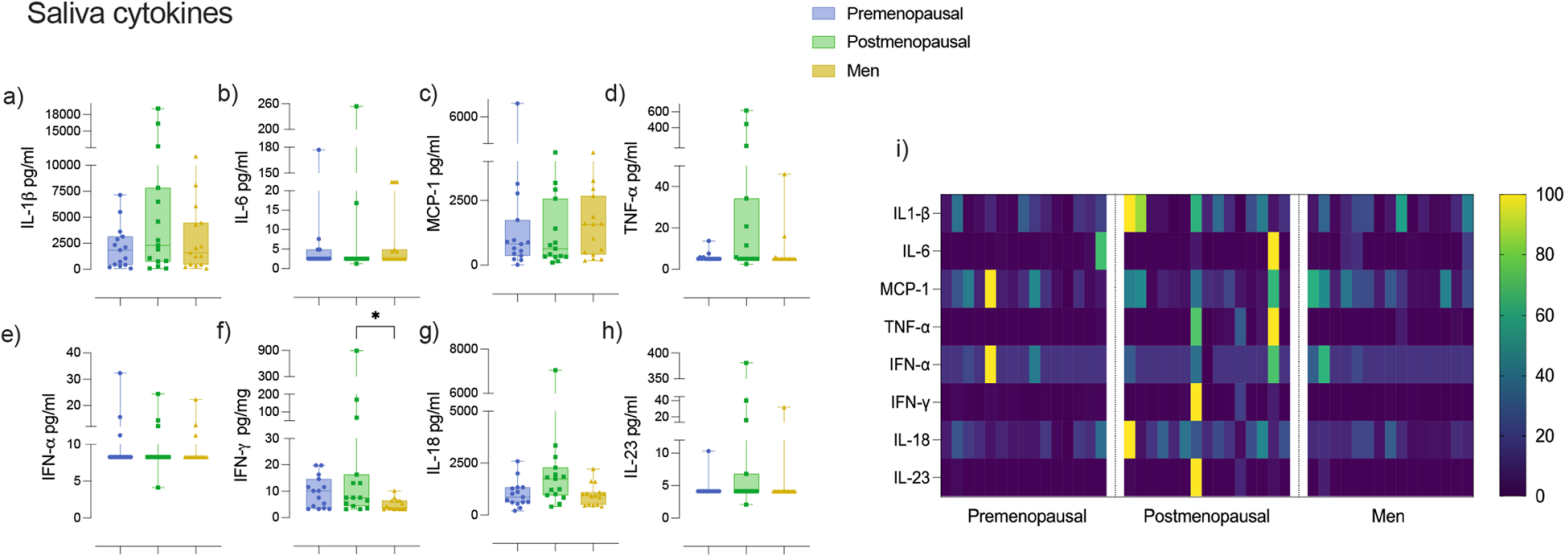
Proinflammatory cytokines in saliva. **a** IL-1β, **b** IL-6, **c** MCP-1, **d** TNF-α, **e** IFN-α, **f** IFN-γ, **g** IL-18 **e**, **h** IL-23 in saliva samples of premenopausal, postmenopausal and male subjects. **i** Heatmap with normalized data of saliva cytokines. Kruskal–Wallis test and Dunn's multiple comparisons test were performed, *p* value less or equal to 0.05 was considered significant. * *p* ≤ 0.05

Key salivary proinflammatory cytokines were measured to evaluate the inflammatory environment of the oral cavity of premenopausal, postmenopausal and male subjects (Fig. 2a-i). Only levels of IFN-γ were significantly higher (*p* = 0.05) in the postmenopausal group compared with the premenopausal and men groups. A similar pattern was observed in IL-1β, TNF-α, IL-18, and IL-23 levels where postmenopausal women had higher levels compared with men and premenopausal but no significance was obtained. In contrast, IL-6 and MCP-1 were slightly higher in men compared with the other groups, with no significance obtained. Overall, this panel reflects a clear tendency of a higher oral proinflammatory state in postmenopausal women (Fig. 2i).

### Oral microbiome signature

**Fig. 3:**
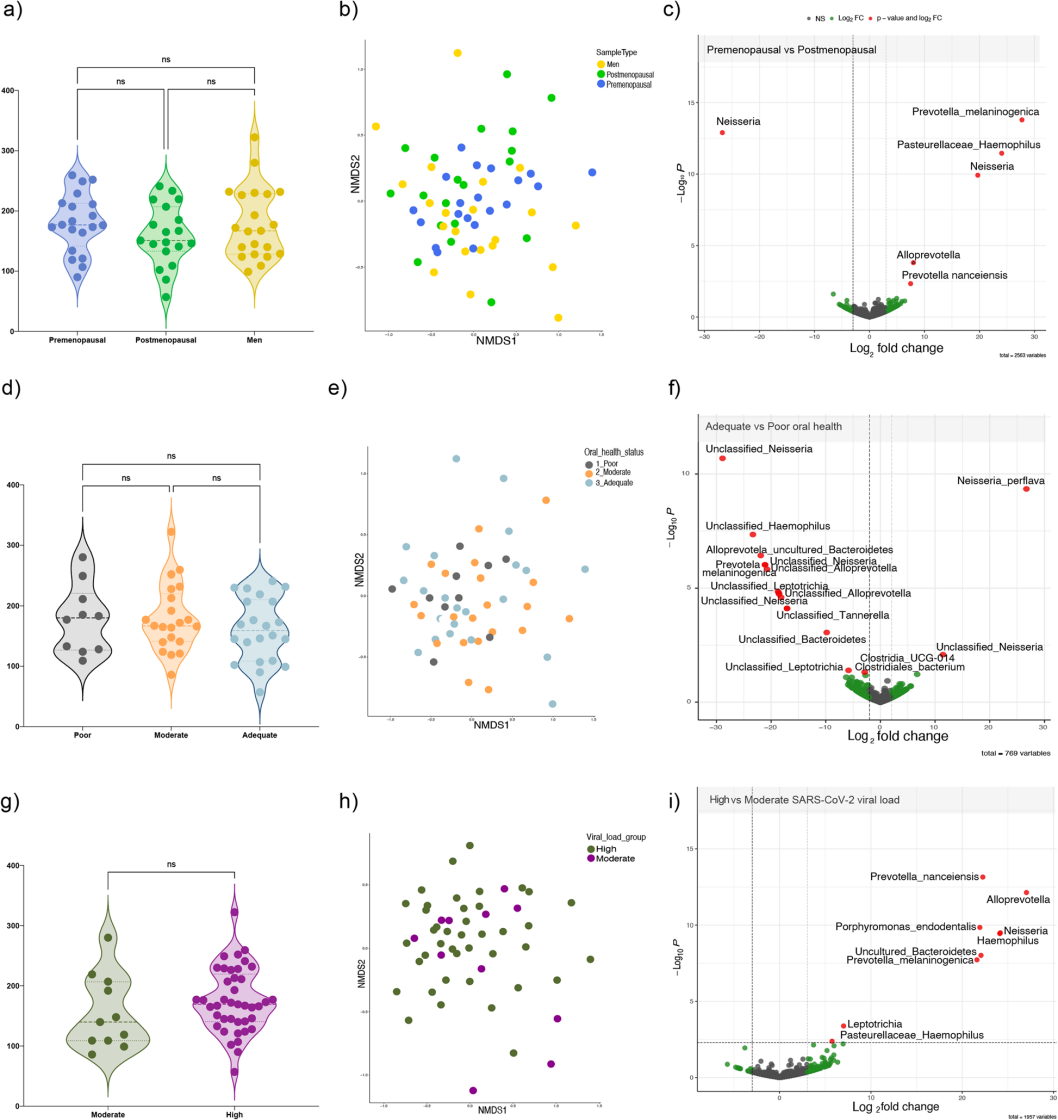
Alpha, beta diversity, and differential abundance of the oral microbiome. **a** Chao1 index, **c** Bray Curtis distance in NMDS plot, **d** Volcano plot based on Deseq2 results of microbiome differential abundance of premenopausal, postmenopausal and male subjects. **d** Chao1 index, **e** Bray curtis distance in NMDS plot, **f** Volcano plot based on Deseq2 results of microbiome differential abundance grouped by oral health status (adequate vs poor). **g** Chao1 index, **h** Bray curtis distance in NMDS plot, **i** Volcano plot based on Deseq2 results of microbiome differential abundance grouped by viral load (high vs moderate). *p* value less or equal to 0.05 was considered significant, and a fold change of 2

In order to describe oral microbiome composition in premenopausal, postmenopausal and men; we performed a metagenome analysis of 16S ribosomal subunit. Alpha diversity was carried out with the Chao1 index, no significant differences were found between the groups. Beta diversity was obtained with nonmetric multidimensional scaling (NMDS) of Bray–Curtis distances; similarly, no significant differences were found between the studied groups (Fig. 3a, b). The tool DESeq2 was employed for the differential abundance analysis, the results were represented in a volcano plot, oral pathogenic bacteria *Prevotella melaninogenica* (Log2 = 26.68; *p* = 1.34e-10)*, Haemophilus* (Log2 = 23.99; *p* = 2.96e-9)*,* and *Alloprevotella* (Log2 = 7.92; *p* = 0.0001) characterized the oral microbiome of premenopausal respect to postmenopausal women (Fig. 3c).

Oral health status can modify the microenvironment; therefore, we re-classify the subjects of the study into Poor, Moderate, and Adequate oral health groups. Strikingly, no differences were found in the alpha and beta indices. Meanwhile, the DESeq2 analysis showed that potential pathogenic bacteria such as *Leptotrichia* (Log2 = -18.74; and *p* = 0.001)*, Tannerella* (Log2 = -17.08; *p* = 0.004)*,* and *Clostridiales* (Log2 = -2.88; *p* = 0.04) represented the poor oral health group compared with the adequate group which was enriched with the commensal microorganism *Neisseria perflava* (Log2 = 26.70; *p* = 1.74 e-7) (Fig. 3d-f).

Subsequently, we grouped the subjects of the study into high and moderate SARS-CoV-2 viral load to evaluate the influence of the viral infection in the oral microbiome. In parallel to the other classifications made, no significant differences were observed in the alpha and beta indices between the groups. As to the differential abundance analysis, only the high viral load group was characterized by *Prevotella nanceiensis* (Log2 = 19.60; *p* = 6.06e-8)*, Prevotella melaninogenica* (Log2 = 21.45; *p* = 9.59e-6)*, Alloprevotella* (Log2 = 23.50; *p* = 2.70e-7)*,* and bacteria from the red complex *Porphyromonas endodentalis* (Log2 = 21.97; *p* = 1.38e-7) (Fig. 3g-i).

### Oral cavity correlations network linkages

**Fig. 4:**
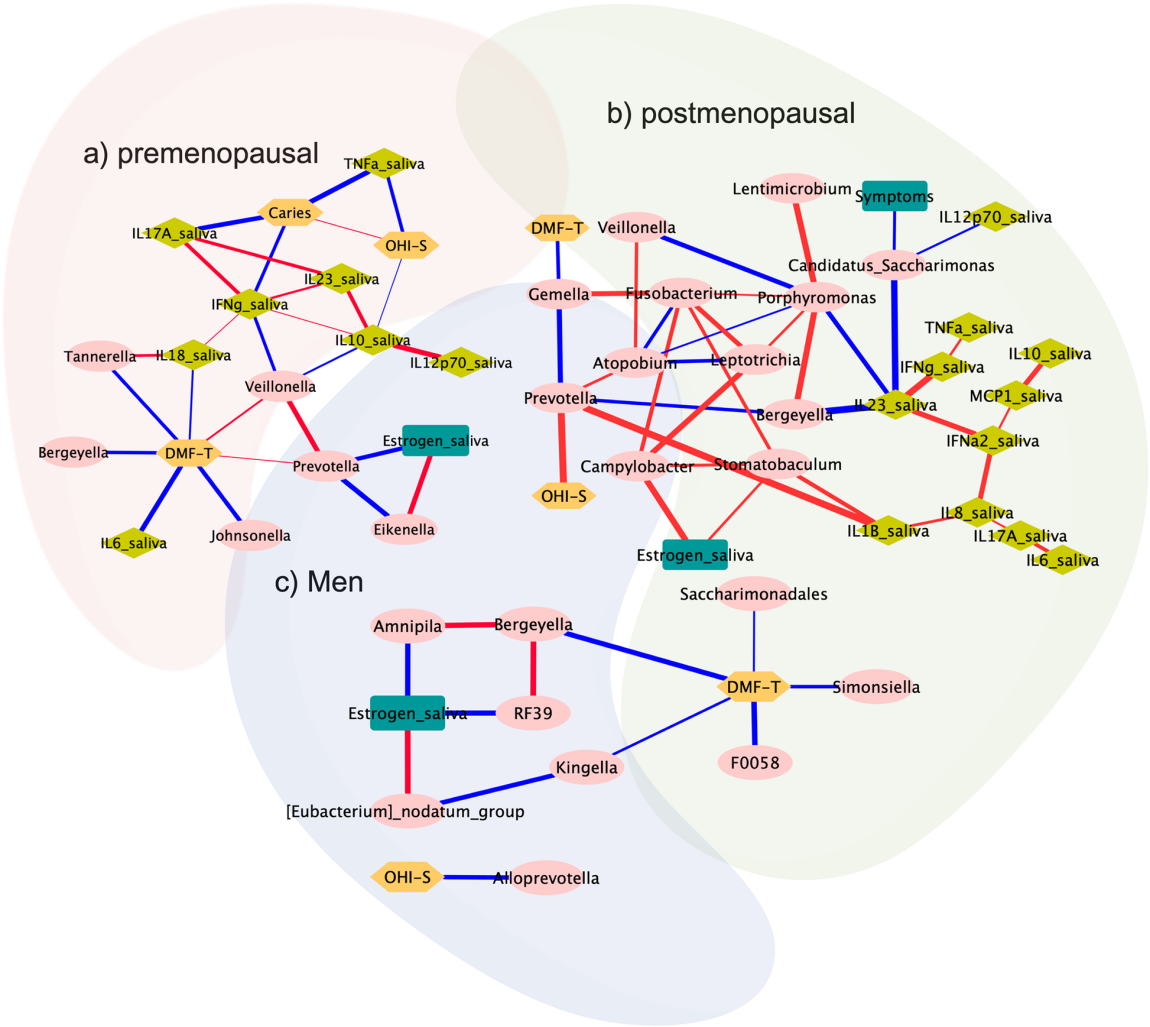
Oral cavity correlations network linkages. **a** premenopausal **b** postmenopausal, **c** Men. Pearson correlation coefficient shows if there exists a positive correlation (red line) or negative correlation (blue line) between variables. *p* value less or equal to 0.05 was considered significant and *r* = 0.4 cut off

Correlation network linkages, for microbiota in saliva with specific key salivary cytokines, estrogen, COVID-19 symptoms, and oral health indices between premenopausal, postmenopausal and men groups are shown in Fig. 4. These correlation networks demonstrated an important interrelation among the studied variables, neither of the groups showed a clear role of salivary estrogens in oral health however oral health indices showed valuable linkages with salivary cytokines and oral bacteria in all of the studied groups.

In the premenopausal group, *Prevotella* and *Veillonella* played a central role in oral health as they were positively correlated with DMF-T (*r* = 0.69; *p* = 0.004; *r* = 0.65; *p* = 0.008, respectively); interestingly, salivary estrogen in this group had a negative correlation with *Prevotella* (*r* = -0.5; *p* = 0.036), which might indicate an indirect hormonal modulation of some proinflammatory oral bacteria. Furthermore, the caries and OHI-S index showed positive correlation (*r* = 0.69; *p* = 0.006), and a negative correlation of IL-10 with the latter and *Veillonella* (*r* = -0.68; *p* = 0.009) (Fig. 4a).

In contrast, postmenopausal was the group with the worst oral health status and was represented by a strong linkage among key proinflammatory oral cytokines. Of note, the oral health index OHI-S showed a positive correlation with the potential oral pathogen *Prevotella* (*r* = 0.52; *p* = 0.049) which along with the caries associated bacteria *Stomatobaculum* had a positive correlation with IL-1β (*r* = 0.61; *p* = 0.023). *Stomatobaculum* was part of a pathogenic triad as it had positive correlation with the pathogens *Fusobacterium* (*r* = 0.59; *p* = 0.016) and *Campylobacter* (*r* = 0.62; *p* = 0.011). Similarly, the cytokines linkages showed positive correlation among each other specially. IFNγ and IL-23 had a positive correlation (*r* = 0.54; *p* = 0.044); as well as between TNF-ɑ and IFNγ (*r* = 0.7; *p* = 0.007). On the other hand, IL-23 had a negative correlation with *Bergeyella* (*r* = -0.54; *p* = 0.048), *Porphyromonas* (*r* = -0.59; *p* = 0.027), and *Candidatus Saccarimonas* (*r* = -0.54; *p* = 0.044), indicative of an immune response and bacterial control (Fig. 4b).

On the other hand, the men group had a reduced number of linkages, however the DMF-T index showed negative correlation with the oral bacterias *Bacteroidetes oral taxon str. F0058* (*r* = -0.54; *p* = 0.042), *Simonsiella* (*r* = -0.6; *p* = 0.02), *Saccharimonadales* (*r* = -0.68; *p* = 0.007), *Bergeyella* (*r* = -0.55; *p* = 0.038), and *Kingella* (*r* = -0.63; *p* = 0.013) from which only the last two have been linked with oral disease (Fig. 4c).

## Discussion

There is sexual dimorphism driven by estrogen in immune responses, as well as in the expression of specific cell receptors such as ACE2, which fluctuate throughout life and is influenced by age, reproductive status, and environmental factors, including the microbiome in the individual [18]. Hence, the oral microbiota, inflammatory response, and clinical effects may be divergent among premenopausal and postmenopausal women and men with SARS-CoV2 infection.

COVID-19 most prevalent oral manifestations reported are dysgeusia and xerostomia; however, mucosal lesions are present in 20% of COVID-19 patients [19]. Poor oral health found in postmenopausal women and men subjects might indicate higher cell damage compared with the premenopausal group. Oral lesions due to SARS-CoV2 infection can be direct or indirect, the latter is related to oral cavity local immune responses, microbial infections, drug side effects among others [19, 20].

ACE2 receptor is highly expressed in the oral mucosa, mainly in the gingival sulcus and the epithelial cells [21, 22], its expression is downregulated in the oral cavity of SARS-CoV2 infected subjects [23]. Here we show that SARS-CoV2-infected postmenopausal and male subjects have lower ACE2 levels; ACE2 downregulation leads to critical inflammatory lesions in the respiratory tract [24]. This downregulation is mirrored in the oral mucosal and partially explains the poor oral health status observed in postmenopausal and male groups compared to premenopausal women.

Oral estrogen decrease impacts the oral mucosa, as it makes it more vulnerable to lesions and infections; additionally, changes the immune system [25] and upregulates ACE2 expression [26]; therefore, representing a protective factor against oral lesions at the onset of the disease [27]. As presumed, we found a negative correlation of viral load with salivary estrogen, which, along with a lower ACE2 level, further denotes the shielding effect of estrogen in the oral cavity in SARS-CoV2 infection.

Salivary proinflammatory cytokines concentrations are increased in COVID-19 [28]; interestingly, in this study, the postmenopausal group had the worst oral health along with a high salivary proinflammatory cytokine profile. A similar pattern was observed in the men group, this effect could be related to the estrogen levels [29]; nevertheless, we could not evaluate this due to the nature of the study.

Oral cytokines and estrogen play a key role in oral microbiome modulation [30, 31]. Oral *Prevotella* species are generally considered pathogenic [32], the premenopausal group was characterized by *Prevotella melaninogenica*, a commensal with potential pathobiont activity [33]. This commensal oral specie has been observed in high proportions in saliva and at the dorsum and lateral sites of the tongue [34]; interestingly, *P. melaninogenica* is able to use estradiol and progesterone as growth factors [35], which could contribute to microbiological homeostasis of the oral niche in the premenopausal women.

Furthermore, postmenopausal correlation linkages show a positive correlation of *Prevotella* with the OHI-S index, indicating a worse oral health status as oral *Prevotella* species increase. Nevertheless, further studies will be needed to elucidate which *Prevotella* species modulate de oral health in postmenopausal women.

*Leptotrichia* and *Tannerella* characterized the poor oral health group; *Leptotrichia* has been isolated and recovered from patients with varying levels of gingivitis [36]. *Tannerella* stimulates the colonization and proliferation of *Porphyromonas gingivalis*, a predominant factor in chronic periodontitis [37]. As a whole, the strong linkage among the proinflammatory species *Tannerella, Porphyromonas, Leptotrichia, Fusobacterium,* and *Stomatobaculum* found in postmenopausal women might indicate a growth support symbiosis net that promotes worse oral health status in this group.

High viral load was mainly distinguished by *Porphyromonas endodentalis*, *Prevotella nanceiensis,* and *Leptotrichia*, reinforcing the hypothesis that viral load is related to a worse oral status and pathogenic pro-inflammatory bacteria colonization. Finally, there is bidirectional communication between oral microbes and the local immune system [30]; this was primarily observed in the postmenopausal group, where pro-inflammatory cytokines clustered together, had strong positive correlations among each other and with keystone pathogenic bacteria such as *Stomatobaculum* and *Prevotella*. Notably, hormonal status during SARS-CoV-2 infection allows us to connect some missing dots to understand oral alterations in the course of COVID-19.

Nevertheless, we are aware that our cross-sectional study has some limitations, like the low number of participants, superficial oral examination, and missing clinical information. Nonetheless, considering the conditions of the COVID-19 pandemic, it was performed in the best possible way. Taking all of these into consideration, our results showed valuable associations, but those cannot reflect causality. Therefore, further functional studies will be needed to deeply understand the role of estrogens in the shaping of oral microbiome, bacterial metabolites and SARS-CoV-2 infection control.

## Materials and methods

### Study population

Sixty non-vaccinated subjects positive to SARS-CoV-2 divided into three groups, premenopausal (*n* = 20), postmenopausal (*n* = 18), and men (*n* = 22) were included in this study. RT-PCR diagnosis was performed by the Laboratorio de Diagnóstico de Enfermedades Emergentes y Reemergentes (LaDEER), Centro Universitario de Ciencias de la Salud (CUCS) Universidad de Guadalajara from april 2021 to january 2022. All participants underwent a questionnaire (S2), oral cavity examination, and blood and saliva sample collection. Inclusion criteria for premenopausal women were age between 18 to 45 years old, and a regular menstrual cycle; in contrast, postmenopausal women were over 45 years old, minimum of six months after the menopausal phase without estrogen replacement therapy. On the other hand, male subjects were over 18 years old. In order to avoid potential sources of bias, all subjects with cancer, autoimmune disease, viral infections (such HIV, HBV, and HCV), hormonal therapy, chronic smokers (more than 5 cigarettes per week), immunomodulatory therapy, oral surgery, antibiotics, prebiotic and probiotics therapy in the last 30 days were excluded from the study.

### Oral status evaluation

Oral indexes were performed to evaluate the oral health status. For the Simplified oral hygiene index (Green and Vermillion index) and Modified Gingival index [38] the evaluated teeth were usually numbered as “16”, “21”, “24”, “36”, “41” and, “44”. To evaluate the presence of caries and the caries history the DMF-T index was used, and it was asked to the patient if there gingival bleeding during toothbrushing. For future analysis the oral status was classified as adequate, moderate and poor. Adequate when there were no cavities hygiene index between 0–1.7 gingival inflammation between 0–1 and no bleeding at toothbrush; moderate when there was 1–2 caries, hygiene index 1.8–2.4 gingival inflammation between 1–2 and no bleeding at toothbrush; finally poor when there were more than two caries, hygiene index higher than 2.5, gingival inflammation between 2–3 and bleeding when toothbrushing.

### Fluorescent immunocytochemistry

Lingual swabs preserved in viral media were realized. Cells extended on slides were performed using shandon clips (cat. 15260, Sigma) at 2,000 rpm in the Cito-Sigma 2.7 (Sigma). Subsequently, cells were fixed with acetone for 5 min, and three PBS washes were realized. PBS-Tween 20 0.2% solution was used for cell permeabilization during 10 min at room temperature. Slides were incubated with BFS 10% solution at 37 ℃ for 1 h, and three PBS washes were realized. Anti-ACE2 (cat. ab282118, Abcam) 1:100 solution was added to slides and incubated in a wet chamber at 4 ℃ overnight. After, three PBS washes were performed, and Alexa fluor 488 (cat. A-11001, Invitrogen) 1:1000 solution was added and incubated at room temperature, protected from light for 2 h. Next to, Vector® TrueVIEW® Autofluorescence Quenching Kit (cat. SP-8400-15, Vector) was added. Subsequently, nucleus stains were done using DAPI (cat. D1306, Invitrogen) 1:5000 solution. Finally, we added Vectashield, all slides were covered with coverslip and sealed with glaze for viewing under the confocal microscope Axio Imager 2 (Carl Zeiss). We used a suspension of endometrial cells as a positive control of ACE2 expression treated with the same protocol that lingual cells. In contrast, the negative control was performed with PBS (without anti-ACE2 antibody) and secondary antibody (Alexa Fluor 488).

### Serum and saliva measurements

Estradiol levels in serum were determined by the chemiluminescent microparticle immunoassay in the ARCHITECT – 1000SR equipment (cat. B7K720, Abbott) following the manufacturing instructions. On the other hand, estradiol in saliva were measured with a high sensitivity Salivary 17-β estradiol ELISA kit (cat. 1–3702, Salimetrics) following the manufacturing instructions, and employing the Multiskan Go microplate spectrophotometer (Thermo Fisher Scientific). Cytokines levels in saliva and serum were performed using a pearl immune assay multiplex (1-Human Inflammation panel 1 [3-plex], LegendPlex cat. 74118, Biolegend) following the manufacturing instructions and reading samples in the Attune NXT Flow Cytometer (Thermo Fisher Scientific).

### 16S rRNA metagenomics

Non-stimulated saliva was collected and mixed with DNA/RNA-Shield (Zymo Research) 1:3 and stored at -80° C. The DNA was isolated with GenElute™ Bacterial Genomic DNA Kit (cat. NA2110, Sigma Aldrich) following the manufacturing instructions. Libraries (V3-V4 16S) were constructed following the workflow of the 16S Metagenomic Sequencing Library Preparation from illumina.

Raw sequencing reads were analyzed on QIIME 2 2023.2 pipeline [39]. The denoise was performed with DADA2 using a quality score of Q20, truncated forward read in 265 nt, reverse reads in 220 nt, and employed a depth of 11,200 reads per sample. Subsequently, sequences were classified in Amplicon Variant Sequence (ASVs) using Silva rRNA Database Project [40]. Shannon and Chao1 indices were used to determine alpha diversity. Bray Curtis distance and PERMANOVA analysis were applied to beta diversity measured. Graphics and statistical analysis were performed with microbiome analyst platform, and Prism.

### Statistical analysis

The online calculator Open-epi was employed, the sample size for comparing two means formula was selected and the calculation was based on the levels of oral estrogen in premenopausal and postmenopausal women. Assuming the reported values we needed 20 subjects for each group with an 80% power and 95% confidence interval. D’ Agostino and Pearson analysis were performed to evaluate the parametric or nonparametric distribution of the data. After that, a Kruskal–Wallis, Chi Square or ANOVA test was performed when appropriate. IF images were obtained from FIJI software version 2.14.0/1.54f. We considered fluorescence intensity sum from 5 Z-stacks per field of analyzed cells. The Prism Graph Pad v. 9.0 program was used for statistical and graphical analysis.

### Supplementary Information

**Additional file 1: S1.** Table of symptoms. **S2.** Questionnaire for demographic and clinical data. **S3.** Table accession numbers to sequences NCBI repository.

## Data Availability

The datasets generated and analysed during the current study are available in the NCBI repository, Submission ID: SUB13844734; BioProject ID: PRJNA1018105, accession numbers are in Table S3.

## Abbreviations

ACE2: Angiotensin Converting Enzyme type 2
IFN-γ: Interferon gamma
IL-1β: Interleukin 1 beta
TNF-α: Tumor Necrosis Factor alpha
IL-18: Interleukin 18
IL-23: Interleukin 23
IL-10: Interleukin 10
MCP-1: Monocyte chemoattractant protein-1
rRNA: Ribosomal ribonucleic acid
PBS: Phosphate Buffered Saline
HIV: Human Immunodeficiency Virus
HBV: Hepatitis B Virus
HCV: Hepatitis C Virus
OHI-S: Simplified Oral Hygiene Index
RT-PCR: Reverse Transcription Polymerase Chain Reaction
DNA: Deoxyribonucleic acid
DMF-T: Decayed, Missing, and Filled primary Teeth
