# Supplementary material for: Insights into estrogen impact in oral health & microbiome in COVID-19
Source: BMC Microbiol. 2024 Jan 20;24:32. doi: 10.1186/s12866-023-03149-5 (PMC10799413; doi:10.1186/s12866-023-03149-5)
Supplement: Supplementary file 1 — Additional file 1: S1. Table of symptoms. S2. Questionnaire for demographic and clinical data. S3. Table accession numbers to sequences NCBI repository. [file 12866_2023_3149_MOESM1_ESM.docx]

**Supplementary material**

**S1. Table Symptoms**

| **Symptoms** | **Premenopausal**  n= 20  (%) | **Postmenopausal**  n=18  (%) | **Men**  n=22  (%) | ***P value*** |
| --- | --- | --- | --- | --- |
| Fever | 9 (45) | 6(33.3) | 6(27.3) | 0.4 |
| Cough | 6(30) | 15(83.3) | 11(50) | 0.004+* |
| Headache | 13(65) | 11(61.1) | 12(54.5) | 0.78 |
| Dyspnoea | 1(5) | 1(5.5) | 1(4.5) | 0.98 |
| Chest pain | 2(10) | 3(16.6) | 2(9.1) | 0.72 |
| Diarrhea | 2(10) | 2(11.1) | 4(18.2) | 0.69 |
| Shaking chills | 4(20) | 1(5.5) | 4(18.2) | 0.40 |
| Odynophagia | 8(40) | 3(16.6) | 6(27.3) | 0.27 |
| Myalgias | 10(50) | 1(5.5) | 6(27.3) | 0.01+ |
| Arthralgia | 8(40) | 3(16.6) | 4(18.2) | 0.16 |
| General malaise | 14(70) | 5(27.7) | 15(68.2) | 0.01+* |
| Dizziness | 3(15) | 2(11.1) | 0 | 0.18 |
| Vomit | 2(10) | 1(5.5) | 0 | 0.32 |
| Rhinorrhea | 5(25) | 2(11.1) | 8(36.4) | 0.18 |
| Conjunctivitis | 5(25) | 0 | 0 | 0.004+ψ |
| Taste disturbances | 9(45) | 8(44.4) | 14(63.6) | 0.41 |
| Anosmia | 10(50) | 5(27.7) | 14(63.6) | 0.07 |
| Number of symptoms |  |  |  |  |
| 1-3 | 3(15) | 4(22.2) | 2(9.1) | 0.51 |
| 4-6 | 13(65) | 12(66.7) | 18(81.8) | 0.41 |
| >7 | 4(20) | 2(11.1) | 2(9.1) | 0.55 |

Comparison by groups: + premenopausal women vs postmenopausal women; ψ men vs premenopausal women; *postmenopausal women vs men

The Chi square test was employed for statistical analysis

**S2. Questionnaire for demographic and clinical data**

1.     What is your name?

2.     How old are you?

3.     How would you describe your gender?

a.      Female

b.     Male

c.      Other

4.     Where are you from?

5.     Where do you live?

a.      Guadalajara

b.     Zapopan

c.      Tlaquepaque

d.     Tonalá

e.      Tlajomulco

f.      Other

6.     Which is your colony and postal code?

7.     What is your highest degree of education you have completed?

a.      High School

b.     Bachelor

c.      Master

d.     PhD

e.      Other

8.     In what sector your professional employment can fit?

a.      Academic

b.     Commercial

c.      Industry

d.     Housekeeping

e.      Unemployed

f.      Other

9.     What is your ethnicity?

a.      Latino or Hispanic

b.     Caucasian

c.      African American

d.     Asian

e.      Other

10.   What is your weight (kg) and height (m)?

11.   What is your blood type?

12.   Do you have a diagnosis of some chronic diseases?

a.      Yes

b.     No

13.   If the answer before was yes; please mention what disease

14.   Do you have a diagnosis of some infectious diseases like HIV, HCV, HBV, Dengue or Influenza?

a.      Yes

b.     No

15.   If the answer before was yes; please mention what disease

16.   Do you have contact with someone positive to COVID-19?

a.      Yes

b.     No

17.   If the answer before was yes; this person was:

a.      In my home

b.     In my work

c.      My partner

d.     My friend

18.   What was the date of contact?

19.   When do you perform your COVID-19 test?

20.   What kind of test do you perform?

a.      Antigen

b.     RT-PCR

c.      Antibodies

21.   This is your first SARS-CoV-2 infection?

a.      Yes

b.     No

22.   When do your symptoms begin?

23.   What symptoms do you present?

24.   Are you an active smoker (5 or more cigarettes per week)?

a.      Yes

b.     No

25.   Do you consume alcohol frequently (5 drinks or more per week)?

a.      Yes

b.     No

26.   Do you consume probiotics, prebiotics or antibiotics in the last 30 days?

a.      Yes

b.     No

27.   Do you consume food supplements in the last 30 days?

a.      Yes

b.     No

28.   Do you consume some prescription drugs daily?

a.      Yes

b.     No

29.   (Only females) Are you pregnant?

30.   (Only females) At what age do you have your first menstrual period (Menorca)?

31.   (Only females) Your menstrual periods are regular?

32.   (Only females) How many pregnancies have you had?

33.   (Only females) At what age do you have the perimenopausal stage?

34.   (Only females) At what age do you have the menopausal stage?

35.   (Only females) Do you have hormonal replacement therapy?

a.      Yes

b.     No

**S3. Table accession numbers to sequences NCBI repository**

| accession | study | bioproject_accession | biosample_accession | sample_name | library_ID | title | platform |
| --- | --- | --- | --- | --- | --- | --- | --- |
| SRR26078937 | SRP461207 | PRJNA1018105 | SAMN37420866 | Premenopausal patient 1 | 93 | 16S RNA seq of human: saliva microbiome | ILLUMINA |
| SRR26078936 | SRP461207 | PRJNA1018105 | SAMN37420867 | Premenopausal patient 2 | 94 | 16S RNA seq of human: saliva microbiome | ILLUMINA |
| SRR26078925 | SRP461207 | PRJNA1018105 | SAMN37420868 | Premenopausal patient 3 | 95 | 16S RNA seq of human: saliva microbiome | ILLUMINA |
| SRR26078914 | SRP461207 | PRJNA1018105 | SAMN37420869 | Premenopausal patient 4 | 96 | 16S RNA seq of human: saliva microbiome | ILLUMINA |
| SRR26078903 | SRP461207 | PRJNA1018105 | SAMN37420870 | Premenopausal patient 5 | 97 | 16S RNA seq of human: saliva microbiome | ILLUMINA |
| SRR26078892 | SRP461207 | PRJNA1018105 | SAMN37420871 | Premenopausal patient 6 | 98 | 16S RNA seq of human: saliva microbiome | ILLUMINA |
| SRR26078881 | SRP461207 | PRJNA1018105 | SAMN37420872 | Premenopausal patient 7 | 99 | 16S RNA seq of human: saliva microbiome | ILLUMINA |
| SRR26078880 | SRP461207 | PRJNA1018105 | SAMN37420873 | Premenopausal patient 8 | 100 | 16S RNA seq of human: saliva microbiome | ILLUMINA |
| SRR26078879 | SRP461207 | PRJNA1018105 | SAMN37420874 | Premenopausal patient 9 | 101 | 16S RNA seq of human: saliva microbiome | ILLUMINA |
| SRR26078878 | SRP461207 | PRJNA1018105 | SAMN37420875 | Premenopausal patient 10 | 102 | 16S RNA seq of human: saliva microbiome | ILLUMINA |
| SRR26078935 | SRP461207 | PRJNA1018105 | SAMN37420876 | Premenopausal patient 11 | 103 | 16S RNA seq of human: saliva microbiome | ILLUMINA |
| SRR26078934 | SRP461207 | PRJNA1018105 | SAMN37420877 | Premenopausal patient 12 | 104 | 16S RNA seq of human: saliva microbiome | ILLUMINA |
| SRR26078933 | SRP461207 | PRJNA1018105 | SAMN37420878 | Premenopausal patient 13 | 105 | 16S RNA seq of human: saliva microbiome | ILLUMINA |
| SRR26078932 | SRP461207 | PRJNA1018105 | SAMN37420879 | Premenopausal patient 14 | 106 | 16S RNA seq of human: saliva microbiome | ILLUMINA |
| SRR26078931 | SRP461207 | PRJNA1018105 | SAMN37420880 | Premenopausal patient 15 | 107 | 16S RNA seq of human: saliva microbiome | ILLUMINA |
| SRR26078930 | SRP461207 | PRJNA1018105 | SAMN37420881 | Premenopausal patient 16 | 108 | 16S RNA seq of human: saliva microbiome | ILLUMINA |
| SRR26078929 | SRP461207 | PRJNA1018105 | SAMN37420882 | Premenopausal patient 17 | 109 | 16S RNA seq of human: saliva microbiome | ILLUMINA |
| SRR26078928 | SRP461207 | PRJNA1018105 | SAMN37420883 | Premenopausal patient 18 | 110 | 16S RNA seq of human: saliva microbiome | ILLUMINA |
| SRR26078927 | SRP461207 | PRJNA1018105 | SAMN37420884 | Premenopausal patient 19 | 111 | 16S RNA seq of human: saliva microbiome | ILLUMINA |
| SRR26078926 | SRP461207 | PRJNA1018105 | SAMN37420885 | Premenopausal patient 20 | 112 | 16S RNA seq of human: saliva microbiome | ILLUMINA |
| SRR26078924 | SRP461207 | PRJNA1018105 | SAMN37420886 | Postmenopausal patient 21 | 113 | 16S RNA seq of human: saliva microbiome | ILLUMINA |
| SRR26078923 | SRP461207 | PRJNA1018105 | SAMN37420887 | Postmenopausal patient 22 | 114 | 16S RNA seq of human: saliva microbiome | ILLUMINA |
| SRR26078922 | SRP461207 | PRJNA1018105 | SAMN37420888 | Postmenopausal patient 23 | 115 | 16S RNA seq of human: saliva microbiome | ILLUMINA |
| SRR26078921 | SRP461207 | PRJNA1018105 | SAMN37420889 | Postmenopausal patient 24 | 116 | 16S RNA seq of human: saliva microbiome | ILLUMINA |
| SRR26078920 | SRP461207 | PRJNA1018105 | SAMN37420890 | Postmenopausal patient 25 | 117 | 16S RNA seq of human: saliva microbiome | ILLUMINA |
| SRR26078919 | SRP461207 | PRJNA1018105 | SAMN37420891 | Postmenopausal patient 26 | 118 | 16S RNA seq of human: saliva microbiome | ILLUMINA |
| SRR26078918 | SRP461207 | PRJNA1018105 | SAMN37420892 | Postmenopausal patient 27 | 119 | 16S RNA seq of human: saliva microbiome | ILLUMINA |
| SRR26078917 | SRP461207 | PRJNA1018105 | SAMN37420893 | Postmenopausal patient 28 | 120 | 16S RNA seq of human: saliva microbiome | ILLUMINA |
| SRR26078916 | SRP461207 | PRJNA1018105 | SAMN37420894 | Postmenopausal patient 29 | 121 | 16S RNA seq of human: saliva microbiome | ILLUMINA |
| SRR26078915 | SRP461207 | PRJNA1018105 | SAMN37420895 | Postmenopausal patient 30 | 122 | 16S RNA seq of human: saliva microbiome | ILLUMINA |
| SRR26078913 | SRP461207 | PRJNA1018105 | SAMN37420896 | Postmenopausal patient 31 | 123 | 16S RNA seq of human: saliva microbiome | ILLUMINA |
| SRR26078912 | SRP461207 | PRJNA1018105 | SAMN37420897 | Postmenopausal patient 32 | 124 | 16S RNA seq of human: saliva microbiome | ILLUMINA |
| SRR26078911 | SRP461207 | PRJNA1018105 | SAMN37420898 | Postmenopausal patient 33 | 125 | 16S RNA seq of human: saliva microbiome | ILLUMINA |
| SRR26078910 | SRP461207 | PRJNA1018105 | SAMN37420899 | Postmenopausal patient 34 | 126 | 16S RNA seq of human: saliva microbiome | ILLUMINA |
| SRR26078909 | SRP461207 | PRJNA1018105 | SAMN37420900 | Postmenopausal patient 35 | 127 | 16S RNA seq of human: saliva microbiome | ILLUMINA |
| SRR26078908 | SRP461207 | PRJNA1018105 | SAMN37420901 | Postmenopausal patient 36 | 128 | 16S RNA seq of human: saliva microbiome | ILLUMINA |
| SRR26078907 | SRP461207 | PRJNA1018105 | SAMN37420902 | Postmenopausal patient 37 | 129 | 16S RNA seq of human: saliva microbiome | ILLUMINA |
| SRR26078906 | SRP461207 | PRJNA1018105 | SAMN37420903 | Postmenopausal patient 38 | 130 | 16S RNA seq of human: saliva microbiome | ILLUMINA |
| SRR26078905 | SRP461207 | PRJNA1018105 | SAMN37420904 | Postmenopausal patient 39 | 131 | 16S RNA seq of human: saliva microbiome | ILLUMINA |
| SRR26078904 | SRP461207 | PRJNA1018105 | SAMN37420905 | Male patient 40 | 132 | 16S RNA seq of human: saliva microbiome | ILLUMINA |
| SRR26078902 | SRP461207 | PRJNA1018105 | SAMN37420906 | Male patient 41 | 133 | 16S RNA seq of human: saliva microbiome | ILLUMINA |
| SRR26078901 | SRP461207 | PRJNA1018105 | SAMN37420907 | Male patient 42 | 134 | 16S RNA seq of human: saliva microbiome | ILLUMINA |
| SRR26078900 | SRP461207 | PRJNA1018105 | SAMN37420908 | Male patient 43 | 135 | 16S RNA seq of human: saliva microbiome | ILLUMINA |
| SRR26078899 | SRP461207 | PRJNA1018105 | SAMN37420909 | Male patient 44 | 136 | 16S RNA seq of human: saliva microbiome | ILLUMINA |
| SRR26078898 | SRP461207 | PRJNA1018105 | SAMN37420910 | Male patient 45 | 137 | 16S RNA seq of human: saliva microbiome | ILLUMINA |
| SRR26078897 | SRP461207 | PRJNA1018105 | SAMN37420911 | Male patient 46 | 138 | 16S RNA seq of human: saliva microbiome | ILLUMINA |
| SRR26078896 | SRP461207 | PRJNA1018105 | SAMN37420912 | Male patient 47 | 139 | 16S RNA seq of human: saliva microbiome | ILLUMINA |
| SRR26078895 | SRP461207 | PRJNA1018105 | SAMN37420913 | Male patient 48 | 140 | 16S RNA seq of human: saliva microbiome | ILLUMINA |
| SRR26078894 | SRP461207 | PRJNA1018105 | SAMN37420914 | Male patient 49 | 141 | 16S RNA seq of human: saliva microbiome | ILLUMINA |
| SRR26078893 | SRP461207 | PRJNA1018105 | SAMN37420915 | Male patient 50 | 142 | 16S RNA seq of human: saliva microbiome | ILLUMINA |
| SRR26078891 | SRP461207 | PRJNA1018105 | SAMN37420916 | Male patient 51 | 143 | 16S RNA seq of human: saliva microbiome | ILLUMINA |
| SRR26078890 | SRP461207 | PRJNA1018105 | SAMN37420917 | Male patient 52 | 144 | 16S RNA seq of human: saliva microbiome | ILLUMINA |
| SRR26078889 | SRP461207 | PRJNA1018105 | SAMN37420918 | Male patient 53 | 145 | 16S RNA seq of human: saliva microbiome | ILLUMINA |
| SRR26078888 | SRP461207 | PRJNA1018105 | SAMN37420919 | Male patient 54 | 146 | 16S RNA seq of human: saliva microbiome | ILLUMINA |
| SRR26078887 | SRP461207 | PRJNA1018105 | SAMN37420920 | Male patient 55 | 147 | 16S RNA seq of human: saliva microbiome | ILLUMINA |
| SRR26078886 | SRP461207 | PRJNA1018105 | SAMN37420921 | Male patient 56 | 148 | 16S RNA seq of human: saliva microbiome | ILLUMINA |
| SRR26078885 | SRP461207 | PRJNA1018105 | SAMN37420922 | Male patient 57 | 149 | 16S RNA seq of human: saliva microbiome | ILLUMINA |
| SRR26078884 | SRP461207 | PRJNA1018105 | SAMN37420923 | Male patient 58 | 150 | 16S RNA seq of human: saliva microbiome | ILLUMINA |
| SRR26078883 | SRP461207 | PRJNA1018105 | SAMN37420924 | Male patient 59 | 151 | 16S RNA seq of human: saliva microbiome | ILLUMINA |
| SRR26078882 | SRP461207 | PRJNA1018105 | SAMN37420925 | Male patient 60 | 152 | 16S RNA seq of human: saliva microbiome | ILLUMINA |
